# N^6^-Methyladenine in Eukaryotic DNA: Tissue Distribution, Early Embryo Development, and Neuronal Toxicity

**DOI:** 10.3389/fgene.2021.657171

**Published:** 2021-05-24

**Authors:** Sara B. Fernandes, Nathalie Grova, Sarah Roth, Radu Corneliu Duca, Lode Godderis, Pauline Guebels, Sophie B. Mériaux, Andrew I. Lumley, Pascaline Bouillaud-Kremarik, Isabelle Ernens, Yvan Devaux, Henri Schroeder, Jonathan D. Turner

**Affiliations:** ^1^Immune Endocrine Epigenetics Research Group, Department of Infection and Immunity, Luxembourg Institute of Health, Esch-sur-Alzette, Luxembourg; ^2^Faculty of Science, Technology and Medicine, University of Luxembourg, Belval, Luxembourg; ^3^Calbinotox, EA7488, Faculty of Science and Technology, University of Lorraine, Vandoeuvre-lès-Nancy, France; ^4^Unit Environmental Hygiene and Human Biological Monitoring, Department of Health Protection, National Health Laboratory (LNS), Dudelange, Luxembourg; ^5^Centre for Environment and Health, Department of Public Health and Primary Care, KU Leuven, Leuven, Belgium; ^6^IDEWE, External Service for Prevention and Protection at Work, Heverlee, Belgium; ^7^Cardiovascular Research Unit, Department of Public Health, Luxembourg Institute of Health, Strassen, Luxembourg

**Keywords:** DNA methylation, 6-methyladenine, embryo development, developmental neurotoxicity, stress, brain

## Abstract

DNA methylation is one of the most important epigenetic modifications and is closely related with several biological processes such as regulation of gene transcription and the development of non-malignant diseases. The prevailing dogma states that DNA methylation in eukaryotes occurs essentially through 5-methylcytosine (5mC) but recently adenine methylation was also found to be present in eukaryotes. In mouse embryonic stem cells, 6-methyladenine (6mA) was associated with the repression and silencing of genes, particularly in the X-chromosome, known to play an important role in cell fate determination. Here, we have demonstrated that 6mA is a ubiquitous eukaryotic epigenetic modification that is put in place during epigenetically sensitive periods such as embryogenesis and fetal development. In somatic cells there are clear tissue specificity in 6mA levels, with the highest 6mA levels being observed in the brain. In zebrafish, during the first 120 h of embryo development, from a single pluripotent cell to an almost fully formed individual, 6mA levels steadily increase. An identical pattern was observed over embryonic days 7–21 in the mouse. Furthermore, exposure to a neurotoxic environmental pollutant during the same early life period may led to a decrease in the levels of this modification in female rats. The identification of the periods during which 6mA epigenetic marks are put in place increases our understanding of this mammalian epigenetic modification, and raises the possibility that it may be associated with developmental processes.

## Introduction

Although it is known to occur in both cytosine and adenine bases, the prevailing dogma is that DNA methylation essentially occurs on the fifth position of cytosine residues. 5-methylcytosine (5mC) is an evolutionarily conserved modification, present throughout both eukaryotes to prokaryotes, which is involved in the development and afterward adaptation to the local environment (LaSalle, 2011; Bonsch et al., 2012; Tobi et al., 2018). Several studies from famine (Roseboom et al., 2011) to stressful events in life, such as war (Trivedi et al., 2019), suggest alterations in the deposition of this modification and its passage to the second and third generation, thereby inducing part of the heritability of certain phenotypes or disorders. Furthermore, early life adversity, such as psychosocial stress, infections or exposure to pollutants, has shown to play a major role in the development of certain disease phenotypes, and DNA methylation is believed to be the link between these events (Murphy et al., 2015; Mitchell et al., 2016; Duca et al., 2018; Elwenspoek et al., 2019; Vonderwalde, 2019). Exposures of rodents to Persistent Organic Pollutants (POPs) through the diet of the dams, such as Brominated flame retardants [e.g., HexaBromoCycloDoDecane (HBCDD) or Polycyclic aromatic Hydrocarbons (PAHs)], during development was shown to induce significant behavioral changes (increased anxious-like behavior, hyperactivity, and altered social behavior) in their offspring (Crepeaux et al., 2014; Maurice et al., 2015). These results underline the existence of a critical window of exposure for brain and behavior development and suggest that epigenetic modifications could be involved in these behavioral impairments (Ibhazehiebo et al., 2011). Furthermore, there is growing evidence that POP exposure might be associated with the occurrence of developmental and neurodegenerative diseases (like Autism, Alzheimer’s and Parkinson’s), through the regulation of DNA methylation, namely 5-methylcytosine (Grova et al., 2019). Although changes in the levels of 5mC has already been linked to POPs (Alvarado-Cruz et al., 2018; Duca et al., 2018), the mechanism behind exposure and later disease development is still unclear.

On the other hand, adenine methylation (6mA) has been known to be present mainly in prokaryotes since it was first described in *E. coli* in 1955 (Dunn and Smith, 1955) and subsequently in *Aerobacter Aerogenes*, *Mycobacterium tuberculosis*, and *Salmonella* (Dunn and Smith, 1958). Since its discovery, 6mA has been associated with important biological processes in bacteria such as DNA replication, regulation of gene expression and cell defense against viruses, through the restriction-modification systems in which DNA adenine methylase (Dam) plays a role (Wion and Casadesús, 2006; Low and Casadesus, 2008; Marinus and Casadesus, 2009; Sanchez-Romero et al., 2015). More recently, adenine methylation was reported in eukaryotic organisms such as plants (Zhou et al., 2018), *Drosophila melanogaster* (Zhang et al., 2015), *Danio rerio* (Liu et al., 2016) and mammals, such as mouse (Wu et al., 2016; Yao et al., 2017; Li et al., 2019) and human (Xiao et al., 2018; Xie et al., 2018). This is currently controversial as studies now suggest that this is due to bacterial DNA contamination in the original eukaryotic samples or that the currently available antibodies also non-specifically bind to unmethylated adenine (Schiffers et al., 2017; O’Brown et al., 2019; Douvlataniotis et al., 2020). Nevertheless, the enigmatic 6mA is particularly interesting in eukaryotes as the reports available so far have associated it with determining the fate of mouse embryonic stem cells, repression and silencing of genes, particularly in the X-chromosome (Wu et al., 2016) and retrotransposons (Li et al., 2020); adaptation to psychosocial stressors (Yao et al., 2017) and tumorigenesis (Xiao et al., 2018; Xie et al., 2018). One common point would appear to be neuronal tissues. Levels of 6-mA were increased in brain tumor biopsies when compared with normal human astrocytes (Xiao et al., 2018). Furthermore, they showed that knockdown of the demethylase ALKBH1, as knockdown, leads to an increase in the proliferation and tumor formation capacity. Adding to this, sequencing analysis showed that gene regions enriched in 6mA were mainly related to neuronal processes (Wu et al., 2016; Yao et al., 2017; Xie et al., 2018). Other studies also reported an increased 6mA brain levels in specific brain areas like prefrontal cortex and amygdala in mice subjected to stress (Kigar et al., 2017; Yao et al., 2017), suggesting a high sensitivity of adenine methylation to several brain insults.

Many of the studies performed so far have relied on 6mA immunoprecipitation and sequencing (MeDIP-seq) to identify the genomic regions susceptible to methylation. A consensus is starting to emerge, focusing on 6mA in LINE-1 elements. Both Yao and Wu reported strong annotations of 6mA in LINE-1 elements in the pre-frontal cortex after stress and embryonic stem cells, respectively (Wu et al., 2016; Yao et al., 2017). Gene ontology analysis showed that in the stress model, 6mA levels negatively correlate with neuronal gene expression and furthermore, the differentially methylated genes overlap with genes present in mental disorders such as depression and autism (Yao et al., 2017) and in the embryonic stem cell model, LINE-1 element methylation impacted their transcription as well as in their neighboring genes (Wu et al., 2016). This is unfortunately contradicted by Xiao who observed significantly higher levels of 6mA in the mitochondria rather than on the genome *per se* (0.18% vs. 0.055%), and Koziol et al. (2016) who reported 6mA in non-coding regions of the genome.

**FIGURE 1:**
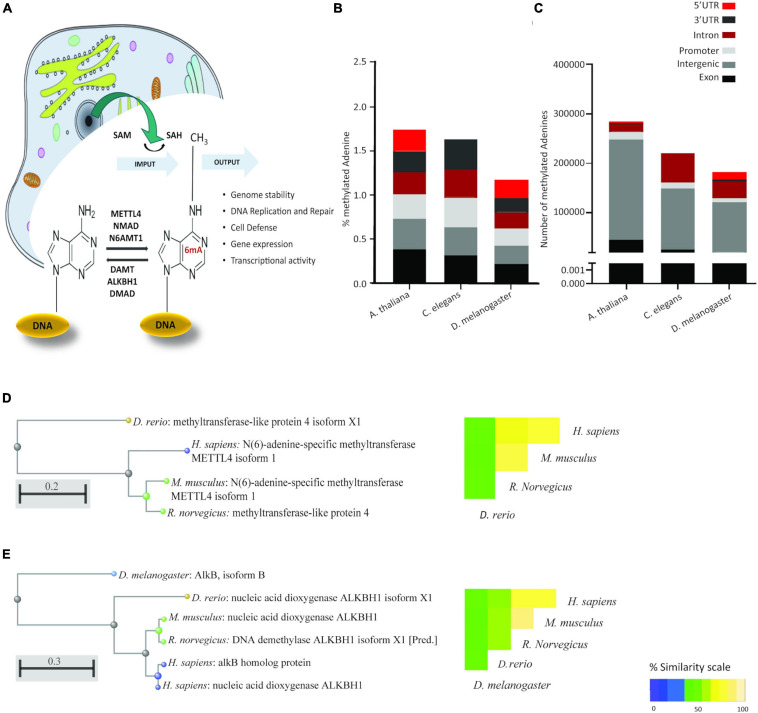
6mA is a conserved eukaryotic epigenetic modification with conserved epigenetic machinery. **(A)** Schematic representation of the current knowledge of 6mA. As for 5mC, the methyl group is donated by S-Adenosyl methionine (SAM), leaving S-Adenosyl homocysteine (SAH). Reported methyl transferases include METTL4, NMAD, and N6AMT1. Demethylases include DAMT, ALKBH1 and DMAD. Cellular compartments are not drawn to scale. **(B)** Relative abundance and **(C)** absolute abundance of direct-read 6mA calls from three eukaryotic species extracted from the MethSMART database of direct calls from PacBio sequencing, with identifiable eukaryotic sequence surrounding the methylation call. **(D)** Hierarchical clustering of sequence alignment and percentage sequence similarity for the methyltransferase METTL4. **(E)** Hierarchical clustering of sequence alignment and percentage sequence similarity for the demethylase ALKBH1.

The data on how adenine is modified and at what point during development or cell-type differentiation the modification is introduced are currently limited. Although the measured levels of 6mA are much lower than 5-mC the available data suggests that 6mA levels increase upon fertilization until the 64-cell stage in zebrafish and then gradually decrease as development progresses. Similarly, in the pig embryo, levels peaked at the morula stage (Liu et al., 2016). The methylase responsible for introducing this modification in eukaryotic species such as man has been reported to be N6AMT1 (Xiao et al., 2018) or METTL4 (Ye et al., 2017; Figure 1A), while the active demethylase has been independently reported twice as ALKBH1 or ALKBH1 in humans (Xiao et al., 2018) and mice, respectively (Wu et al., 2016). Elegant over-expression and knockout studies of both these enzymes, have demonstrated that a reduction in 6mA levels promotes tumorigenesis (Xiao et al., 2018) whilst increasing 6mA leads to inhibition of glioblastoma formation (Xie et al., 2018).

In this study, we evaluate the levels of 6mA in different eukaryotes species, with a special focus on mammals. Although previous studies affirm that this adenine modification is present in eukaryotes at a low density, we demonstrate that is it widely present in zebrafish, mice, rat, and human, specifically in the brain. Furthermore, we report that 6mA steadily increases during embryogenesis in both zebrafish and mice and its global levels show subtle changes in the cerebellum of female rats upon exposure to the brominated flame retardant α-HBCDD during early life. Collectively, these results may help shed a light on our understanding of the mammalian epigenetic modifications.

## Materials and Methods

### Identification of 6mA From Existing Sequencing Results

The MethSMRT database of 6mA signals in single-molecule real-time (SMRT) sequencing was interrogated (Ye et al., 2017). MethSMRT contains single-nucleotide resolution of 6mA throughout many genomes extracted from all publicly available PacBio SMRT sequencing. For the worm (***Caenorhabditis elegans***), brewer’s yeast (***Saccharomyces cerevisiae***), thale cress (***Arabidopsis thaliana***) and the fruitfly (***D. melanogaster***) the numbers of unmodified adenine residues, 6mA residues and their genomic locations were extracted. Protein sequences from man (***Homo***sapiens****), Norway rat (***Rattus***norvegicus****), house mouse (***M***usmusculus****), zebrafish (***Danio***rerio****), and the fruitfly (***Drosophila***melanogaster****) were extracted from the NCBI under accession numbers NP_006011.2, NP_001102188.1, NP_001096035.1, NP_001018527.1, and NP_996458.1, respectively, for ALKBH1. For METTL4, accession numbers used for man, Norway rat, house mouse and zebrafish were NP_073751.3, NP_795891.2, NP_001178743.1, and XP_689178.3, respectively. Sequences alignment and phylogenetic trees were obtained with COBALT, a multiple sequence alignment tool from NCBI (Papadopoulos and Agarwala, 2007; Madeira et al., 2019). Genotype-Tissue Expression data for human METLL4 and ALKBH1 enzymes described in this manuscript were downloaded from the GTEX portal on August 19th, (dbGaP Accession phs000424.v8.p2, 19/08/2020).

### Zebrafish Embryos

Fish were housed in a ZebTEC standalone recirculating tank system (Techniplast, Buguggiate, Italy) kept at 28°C on a light:dark (14:10 h) cycle. Fish were fed twice per day: once with granular food (Special Diet Services, Essex, United Kingdom) and then with freshly prepared brine shrimp (*Artemia salina*). Male and female adult wild-type AB fish were mated and their offspring collected immediately following fertilization. Zebrafish embryos were dechorionated via a 5 min incubation with pronase (2 mg/mL diluted in E3 medium; Roche Diagnostics GmbH, Germany) before being washed and raised in E3 medium (0.33 mM CaCl2, 0.17 mM KCl, 5 mM NaCl, 0.33 mM MgSO4, and 0.1% Methylene Blue, pH 7.4) as previously described (Ernens et al., 2017). To ensure sufficient genetic material for DNA extraction, zebrafish embryos were pooled at different densities across the selected time points as follows; 100 embryos were pooled immediately after fertilization (0 h post fertilization), 30 embryos at 24 hpf (hours post fertilization) and 20 embryos at 48, 72, and 120 hpf. Four biological replicates were collected at each time point.

### Mice Embryos

Female Balb/c mice were group housed (*n* = 5 per cage) for 10–14 days for Lee-Boot cycle synchronization and brought into estrous by exposure to soiled bedding from a male mouse. Two females in estrous and one male were placed in a clean “breeding” cage and left together overnight. Mating was confirmed by the presence of a vaginal plug and all animals were returned to their home cage until sacrifice. Pregnant females were sacrificed at six different time-points, from E7 to E21. The uterus was extracted and placed in 1X PBS (4°C) in a petri dish. Embryos were removed from the uterus, decapitated and the brain dissected. Hippocampi was dissected and stored at −20°C until further analysis.

### Post Mortem Tissue From Human Brains

Human material used in this study is from a previously reported study (Cao-Lei et al., 2013). Briefly, tissues were collected from five different donors from the region of Chang Mai, in Northern Thailand. The subjects had no underlying diseases and were hospitalized due to either car accidents, blunt chest injury, or gunshot. All patients died in the Chiang Mai University Medical Hospital, where the autopsies were carried out 2–10 h after the death. A 5 mm punch biopsy was collected from the initial one-centimeter sections, for each of the 28 different brain regions.

### Perinatal Exposure to the Brominated Flame Retardant α-HBCDD (HexaBromoCycloDoDecane) as an Environmental Early Life Adversity

As previously reported (Maurice et al., 2015), pregnant females Wistar dams were exposed daily to the α isomer of HBCDD for 42 days by receiving a volume of α-HBCDD contaminated eggs at different concentrations (0, 22, and 66 ng/kg/day), from gestational day (GD0) onto the weaning of the offspring (PND21), constituting a solid early life adversity model in lab rats (Sengupta, 2013). Concentrations were calculated according to the human exposure through egg consumption and 22 and 66 ng/kg/day were found to correspond, in rats, to the lowest and highest levels of human contamination from eggs (Koch et al., 2015).

### Behavior Analysis

At PND270, sensory and motor impairments were evaluated by using the Locotronic apparatus (Locotronic Intellibio, France), according to the manufacturer’s instructions. The later was composed of a starting and arrival box, connected by a horizontal ladder corridor, with 3 mm diameter bars (7 mm spacing). Two trials were run and infrared sensors above and below each inter-bar space read the position, number, and duration of missteps of the animals. Offspring were sacrificed at PND270.

### DNA Extraction

DNA was extracted from samples through a spin-column procedure, using the QIAamp DNA Micro Kit (Qiagen, Hilden, Germany), following the manufacturer’s instructions. DNA quantity and quality were assessed with both nanodrop (Thermo Fisher Scientific, Belgium) and Qubit (Thermo Fisher Scientific).

### 6mA Detection by HPLC MS/MS

Prior to LC-MS/MS analysis, isolated genomic DNA was enzymatically hydrolyzed to individual deoxyribonucleosides in a one-step procedure. Sample DNA (1 μg) was supplemented with 2.5 ng [15N3]-2′-deoxycytidine (Cambridge Isotope Laboratories, Inc., France) as internal standard, dried under N2 and then hydrolyzed at 37°C, in a 10 μl digestion mix of phosphodiesterase I (300 mU; Sigma-Aldrich, France), alkaline phosphatase (200 U; Sigma-Aldrich), and Benzonase® Nuclease (250 U, Sigma-Aldrich) in Tris buffer pH 7.9 (20 mM Tris; Sigma-Aldrich), for about 8 h (Godderis et al., 2015). In each sample, DNA 6-methyl-adenine was determined as previously published (Cardenas et al., 2017; [Bibr B8][Bibr B11]) with minor modifications: After hydrolysis, samples were diluted with water (500 μL), filtered using an Amicon Ultra-0.5 Centrifugal filter device (Sigma-Aldrich) and resuspended in a solution of ACN: H_2_O (70:30, v/v). An aliquot of 10 μL was injected on a hydrophilic interaction liquid chromatography (HILIC) column (Acquity UPLC BEH Amide columns 1.7 μm, 2.1 × 50 mm; Waters Corp.), held at a temperature of 40°C. A mixture of 1 mM Ammonium Fluoride (A) and acetonitrile (B) was used as the mobile phase for chromatographic separation. A flow rate of 0.4 mL/min was applied. All HPLC solvents and reagents were from Sigma-Aldrich (LC-MS/MS grade). The analyses were carried out using a Waters Xevo TQ-XS triple quadrupole mass spectrometer (Wexford, Ireland) with an electrospray ionization source (ESI) in positive mode. Multiple reaction monitoring (MRM) with an argon collision gas was used to improve quantification, selectivity and sensitivity. MS/MS parameters for the specific detection by MRM are detailed in Table 1. The peaks were identified as previously described (Grova et al., 2020). Samples have been analyzed in a random manner and without prior identification of the different treatment groups.

**TABLE 1:** MS/MS parameters for MRM detection of modified and unmodified hydrolyzed nucleosides.

**Compounds cone**	**Ionization mode**	**Transitions (m/z)**	**Collision energy (eV)**	**Cone (V)**
-2′-deoxycytidine (IS)	ESI+	231 → 115	15	12
2′-deoxyadenine	ESI+	268 → 135	18	26
	ESI+	268 → 119	42	26
N^6^-methyl-2′-deoxyadenine	ESI+	282 → 149	14	2
	ESI+	282 → 133	40	2

### Oligonucleotide Synthesis

A spike-in sequence of 201 base pairs was inserted in a pUC57 plasmid backbone of 2,710 base pairs, with *Eco*RV producing a final product of 2,911 base pairs (GeneCust, Boynes, France). The spike-in sequences were carefully designed to have 10 GATC sequences along the 201 base pairs, which is the preferred motif for dam methyltransferase when modifying the adenine residues (Supplementary Figure 3B). The sequences were later amplified with the following primers: forward 5′-GCCTCGTGAAATCCCGTTAG-3′ and reverse 5′-TGAAGGTGCCAAGAAGTTTCC-3′. The PCR products were then treated with dam methyltransferase for synthesis of dot blot positive (loading) control.

### Enzymatic Treatments of DNA for Generation of Positive Controls

Non-methylated Yeast DNA was used as a negative control, and artificially methylated DNA was used as a second positive control. To generate it, 2 μg of Yeast DNA were incubated together with nuclease free water, 10X reaction buffer, S-Adenosyl methionine (160 μM) and 2 μL of EcoGII enzyme (NEB labs, Frankfurt, Germany), for 4 h at 37°C. Similarly, 1 μg of PCR product of the created oligonucleotide was incubated with nuclease free water, 10X reaction buffer, S-Adenosyl methionine (160 μM) and 1 μL of dam methyltransferase enzyme (NEB labs), for 4 h at 37°C. Both reactions were heat-inactivated at 65°C, for 15 min. Signals were detected (Intas ECL Chemocam Imager) after 5 min incubation with ECL Plus Western Blotting Substrate (Pierce; Thermo Fisher Scientific) following the manufacturers’ recommendations. Signal intensity was quantified with ImageJ software (Schneider et al., 2012).

### Dot Blot

Immunoblotting was performed and normalized as previously described (Wu et al., 2016; Xie et al., 2018; Kweon et al., 2019). Briefly, DNA was denatured at 95°C for 10 min, flash-cooled on ice and neutralized with 10% (v/v) of 6.6M Ammonium Acetate. Samples (40 ng, 4 μl per sample) were spotted on a nylon membrane (Whatman Nytran SuperCharge; Sigma), air-dried for 10 min and UV-crosslinked for 90 s (InGenius syngene bio imaging). Membranes were then blocked (5% non-fat milk, 1% BSA in 0.1% PBST) for 1 h at room temperature (RT) followed by incubation with the primary anti-N6-methyladenine antibody (Synaptic Systems) diluted 1:1000 in blocking solution overnight, at 4°C. After washing three times with 0.1% PBST, membranes were incubated with anti-rabbit IgG antibody (Thermo Fisher Scientific) diluted 1:5000 in blocking solution for 2 h at RT.

### Immunohistochemistry

At PND270, brains from the dams of the animals treated with α-HBCDD were excised, flash frozen, and stored at −80°C until analyzed. For IHC analysis, serial sections (20 μm) were mounted on Super Frost slides (Roth Sochiel, Lauterbourg, France).

#### 6mA

After temperature equilibration (10 min, RT), slides with cerebellum sections from two female and two male individual rats were rinsed with PBS 1x for 5 min, and underwent rehydration and permeabilization (PBS1X, Triton-X 0.3%), for 10 min. Slides were subjected to antigen retrieval (2M HCl) for 45 min and neutralization (0.1M Tris–HCl) for 20 min. After blocking for 1 h (PBS1x, BSA 1%, Triton-X 03%, RNAse A 50 μg/mL), slides were washed with 1X PBS (3 × 5 min) and incubated with primary antibody (1:500, rabbit-anti-6mA; Synaptic Systems, Gottingen, Germany) for 1 h (RT). After washing (3 × 5 min, 1x PBS) a 1:2000 Alexa Fluor 488-anti-rabbit IgG (ab150077; Abcam, Cambridge, United Kingdom) was used for visualization. Finally, the slides were washed with 1X PBS (3 × 5 min) and mounted with anti-fade mounting medium which contained DAPI (Thermo Fisher Scientific).

#### Cytochrome c Oxidase

Cerebellum sections were incubated with a cytochrome substrate buffer [100 mg DAB-4HCl (Sigma-Aldrich), 40 mg cytochrome c (Sigma-Aldrich), 36 g catalase (Sigma-Aldrich) in 0.1 M Phosphate buffer, pH 7.4] for 65 min at 37°C, in the dark and with agitation, as previously described (Crepeaux et al., 2014). The reaction was stopped by washing the sections for 5 min with cold 0.1 M Phosphate buffer containing 10% sucrose. Sections were then fixed in 4% formaldehyde for 30 min, washed three times with 0.1 M Phosphate buffer and dehydrated with ascending ethanol baths (50, 70, 96, and 100%) for 3 min. Finally, sections were cleared with toluene twice for 5 min and mounted with Eukitt mounting medium (Sigma-Aldrich). Stained sections were analyzed with a BIOCOM system (Les Ulis, France), using the standard curve to calculate μmol/min/g of tissue from optical density.

### Statistical Analysis

All results were expressed as mean ± SEM. For each group, a Kolmogorov–Smirnov test for normality of the distributions as well as a Bartlett test for equality of variances were performed. When normality of the distribution and homogeneity of variance were assumed, a two-way ANOVA test was performed to compare different groups, followed by a *post hoc* Bonferroni *t*-test. Main effect and interaction effects are reported. Locomotor activity in the Locotronic apparatus was analyzed using non-parametric procedures (Kruskal–Wallis test for comparisons among the three groups at the 1st and the 2nd trials, Wilcoxon procedure to compare performances between the two trials in each group). Statistical analyses were carried out using SPSS 16.0 software (SPSS Inc., Chicago, IL, United States) or GraphPad Prism version 8.0.0 for Windows (GraphPad Software, San Diego, CA, United States). Heatmaps were generated in R v3.5.3.

## Results

### 6-mA as a Genuine Eukaryotic Modification

**FIGURE 2:**
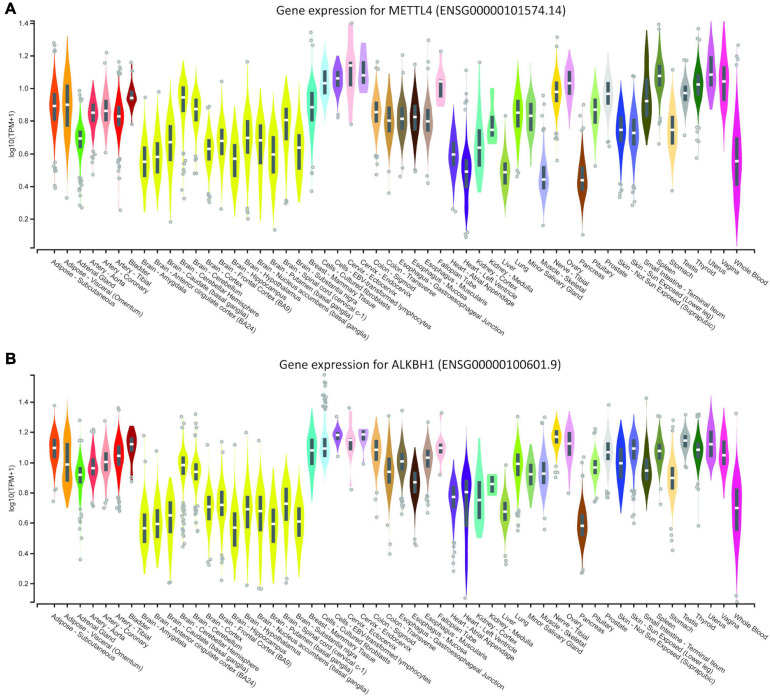
Genotype-Tissue Expression data for **(A)** human METLL4 methyltransferase and **(B)** ALKBH1 demethylase. Data were downloaded from the GTEX portal on August 19th, (dbGaP Accession phs000424.v8.p2, 19/08/2020).

As the literature casts doubt on the existence of 6mA in eukaryotic DNA, we searched the MethSMRT database for direct 6mA reads from PacBio SMRT sequencing data. Direct reading of 6mA from the SMRT data ensures that the DNA from the target species is genomic DNA and does not represent contamination from bacterial, viral or mitochondrial DNA. 6-mA levels in three different species, *D. melanogaster*, *C. Elegans*, and *A. thaliana* (Figure 1B) are in accordance with the literature, ranging from 1.25 to 1.75% of the total existent Adenines, representing 200,000 to 290,000 adenines in each of the genomes (Figure 1C). This modification appears to be more abundant in Exons and Promoter regions in all three species, expanding the observation in human cell lines and in *D. melanogaster* (Xiao et al., 2018; Yao et al., 2018) to *A. thaliana* and *C. elegans*. Furthermore, homologs of three key enzymes in the methylation/demethylation machinery were identified in *H. sapiens*, as well as *D. rerio*, *M. musculus*, and *R. norvegicus* (Figures 1D,E). ALKBH1 was also present in *D. melanogaster* (Figure 1E). METTL4 is a conserved methyltransferase, as well as N6AMT1, while ALKBH1 is a conserved demethylase. The sequences of these were highly conserved with an average 71% similarity between species (range 42–92%; Figures 1D–E). Available RNA Seq data from the GTEXx and Illumina human body map v2.0 suggests that both ALKBH1 and METTL4 enzymes are expressed throughout the human body and that levels are uniform. We investigated the linear correlation between the tissue expression of both enzymes and discovered that they are similarly expressed in all tissues suggesting that a defined level is required in all tissue of the body, either for housekeeping purposes or as a mechanism to ensure correct functioning of the 6mA machinery (Figures 2A,B and Supplementary Figure 1A). N6AMT1 does not show such a high correlation with ALKBH1 as METLL4 (Supplementary Figure 1B), but shows an equally high tissue distribution and interspecies similarity (Supplementary Figures 2A,B).

### 6-Methyladenine Detection and Quantification

**FIGURE 3:**
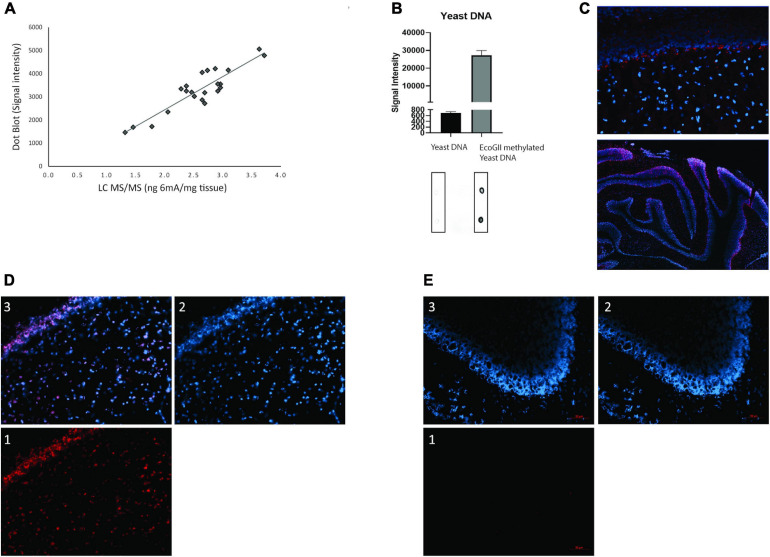
**(A)** Representation of the linearity between the dot blot and LC-MS/MS techniques (linear relationship was evaluated by Spearman analysis, *n* = 26, *r*^2^ = 0.81, *p* < 0.001); **(B)** Quantification of positive and negative control dot blots with a representative membrane underneath; **(C)** Immunofluorescence 6mA detection in cerebellum of female and male rats exposed through the dams to α-HBCDD at PND270. DAPI in blue, primary AB anti-6mA in red; **(D)** Immunofluorescence 6mA detection separated into (1) red channel – 6mA, blue channel – DAPI (2) and merged channels (3); **(E)** background immunofluorescence separated into (1) red channel – secondary antibody only, blue channel – DAPI (2) and merged channels (3). All images except C upper: 20x objective. C upper: 60x objective. In all images red scale bar 50 μm.

We established 6mA detection using two independent techniques. Initially we established a LC-MS/MS detection of 6mA with increased selectivity since in addition to compound identification with retention time; we were also able to confirm its presence with MRM (multiple reaction monitoring) transitions. The analytical method is detailed in Table 1. Furthermore, an extra confirmation transition was used to ensure the presence of each target compound. To confirm the later, the ratio “quantification transition to confirmation transition” was determined as the difference from the ratio obtained with standard compounds below 20%. Based on previous literature reports (Wu et al., 2016; Xiao et al., 2018), we also measured 6mA using dot blot with a specific antibody against 6-methyladenine (6mA). This technique allowed us to measure the levels of this modification through quantification of the signal intensity of the DNA dots spotted on a membrane given by the oxidation of the luminol present in the revealing agent. The linearity of detection between the dot blot and LC-MS/MS techniques was then evaluated to validate the presence of 6mA in eukaryotic DNA (Figure 3A). DNA samples, isolated from cerebellum collected from both female and male rats exposed or not to α-HBCDD at 22 and 66 ng/kg/day, was subjected to both types of analysis. Figure 3A displays a linear correlation between the two sets of data (Spearman analysis, *n* = 26, *r*^2^ = 0.81, *p* < 0.001) confirming the suitability of these two methods to produce comparable results.

### 6mA Antibody Cross-Reactivity

In order to have suitable positive controls, we tested the cross-reactivity of the 6-methyladenine antibody (synaptic systems) with in-lab methylated Yeast DNA and a methylated oligonucleotide. As seen in Figure 3B and Supplementary Figure 3A, Yeast DNA is devoid of 6mA and when treated with EcoGII, the antibody clearly detects a 40-fold increase of 6mA. For the methylated oligo, when treated with dam methyltransferase, the antibody clearly detects a higher signal (left) when compared to the non-treated PCR product (right). Based on this, all further 6mA results presented in this study are based on dot blot analysis. Finally, by using immunochemistry, we visualized for the first time the presence of 6mA in the cerebellar cells of rats (PND270) exposed or not through the dam to α-HBCDD at 66 ng/kg/day. Methylation of adenine (in red) is shown to be nicely located in the nucleus of cerebellar cells (in blue) (Figure 3C). Figures 3D,E are, respectively, representative images of control and treated animals, divided into the different channels, with the same settings, demonstrating that the control group has no 6mA background (Figures 1, 3E). As only two individuals/sex/group were taken to generate the present images, no quantification of the signal was done.

### 6-Methyladenine Presence in Mammals

**FIGURE 4:**
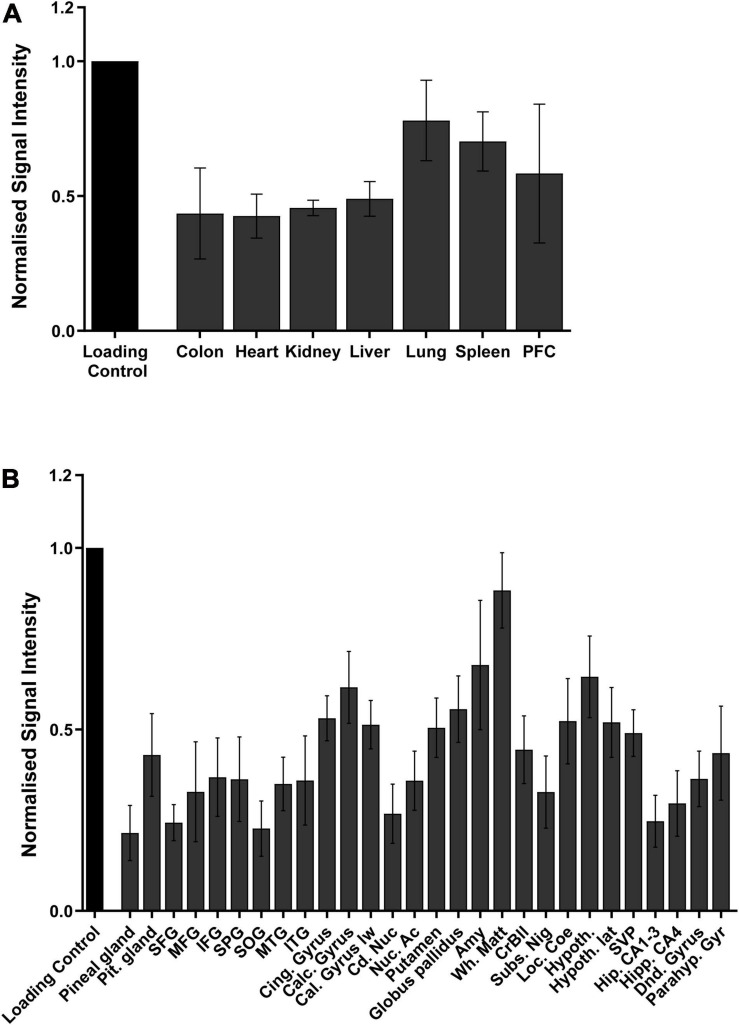
Quantification of the distribution of 6mA throughout the different tissues in **(A)** mice and in **(B)** different human brain regions, via dot blot. Data are from four biological replicates in mice and five in humans, and presented as mean ± SEM.

Having convinced ourselves that 6mA was a genuine eukaryotic epigenetic modification that we could detect, we determined its body-wide tissue distribution in a model species, the mouse, and performed a detailed examination in the most relevant human tissue. DNA from nine different adult mice organs, from the cardiovascular to the neuronal and digestive system, was examined by dot blot for 6mA. This modification was detected in all organs and its abundance, unlike the classical 5mC, was not equally distributed throughout the tissues. The highest abundance was in the lung, spleen and brain, represented by prefrontal cortex (PFC), (Figure 4A) that were 1.8, 1.7, and 1.9 times more abundant than the weakest tissue: the heart. The higher levels in the brain appears to agree with the GTEx RNA-Seq data that suggests lower expression of the demethylase enzymes in the brain. As such, we performed a detailed examination of 6mA presence in twenty-eight different human brain regions from five human donors. Additionally, data from the human brains confirm this abundance where tissue specificity also seems to play a role with white matter being the most methylated tissue, having 3.9 times higher levels of 6mA then the weakest tissue: the Superior occipital gyrus (SOG) (Figure 4B). The amygdala is the second brain region with higher methylation levels, having almost three times more methylated adenines than the SOG. This is a region involved in fear conditioning and known to be susceptible to stressful events and, together with our previous findings, these observations consolidate the fact that 6mA may play a role in the response to stress.

### Embryonic States of Both Mice and Zebrafish Show a Linear Increase in the Levels of 6mA

**FIGURE 5:**
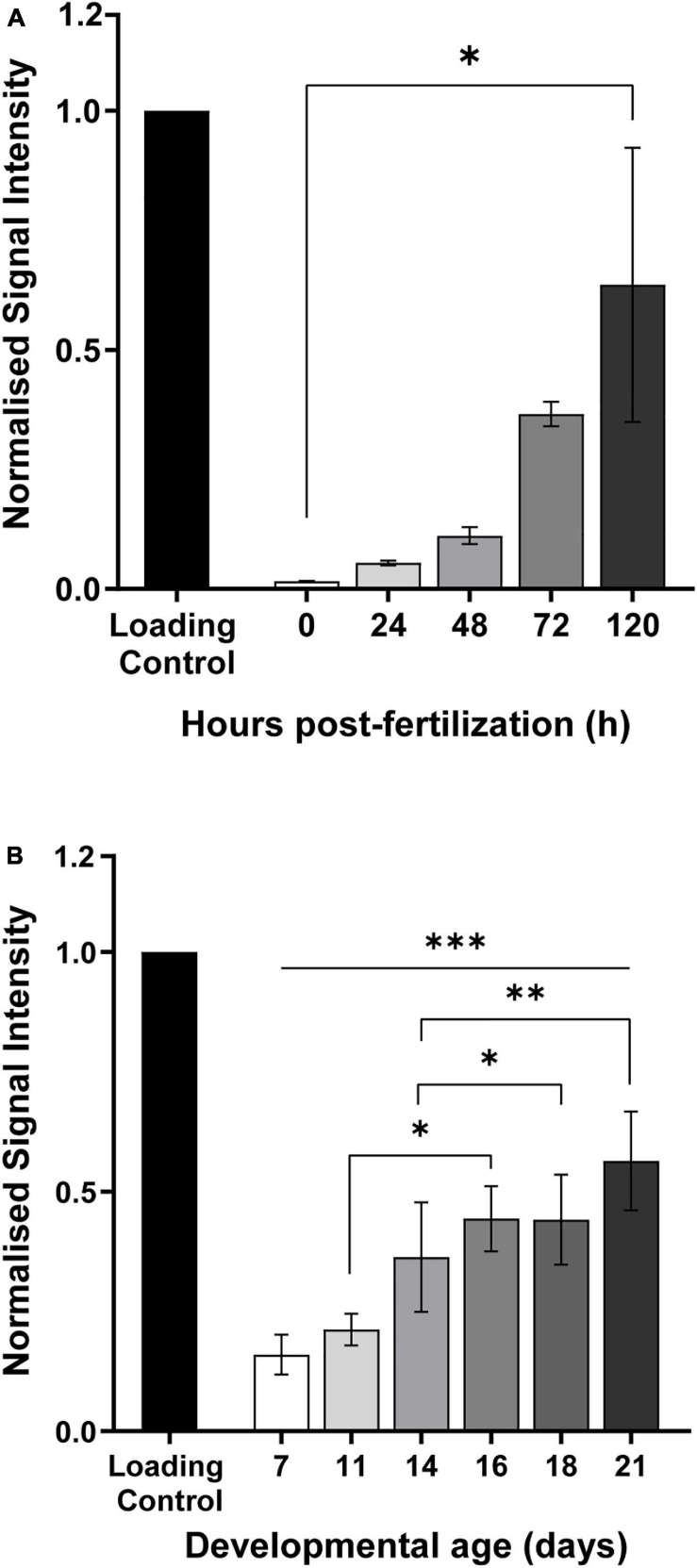
Quantification of 6mA modification in genomic DNA extracted from **(A)** zebrafish and **(B)** mice, at different developmental stages. Later embryonic stages show a higher accumulation of this modification when comparing to the early stages. Data are from 20 to 100 biological replicates for zebrafish and 5 to 8 for mice, and presented as mean ± SEM. *p < 0.05; **p = 0.001; ***p = 0.0003.

Mice and zebrafish embryos were collected at different time points, in order to cover the whole gestational period, a critical period in the fetal development. Contrarily to 5mC, dynamic changes throughout early and late development, adenine modifications seem to steadily increase as the embryo develops from a single pluripotent cell to an almost fully formed individual consisting of mainly somatic cells, both in zebrafish and mice (Figures 5A,B). In mice, from day 7 to a fully formed embryo the amount of 6mA significantly increases 3.5 times (^∗∗∗^*p* < 0.001) and the same trend can be observed throughout the pregnancy with significant increases of 1.8 times from day 11 to 16 (^∗^*p* < 0.05) and 1.6 times from day 14 to 16 (^∗^*p* = 0.01) (Figure 5A). In zebrafish, the same behavior is observed and although is not significantly increasing during the development process, the values of 6mA suffer a 38 fold increase from fertilization to fully formed embryo (^∗^*p* < 0.05) (Figure 5B). These observations suggest that 6mA may play an important role in the development and can help us understand how a deviation from normal development can trigger the disease phenotypes such as neurodevelopmental disorders later in life.

### Early Life Exposure to α-HBCDD Induces Change in 6mA Levels in F1 Generation at PND270

After demonstrating the steadily increase of 6mA throughout development, we evaluated how values of 6mA change in the brain of rats followed by exposure to α-HBCDD through the dams in a known neurodevelopmental toxicity model. There was a trend toward lower 6mA levels 9 months after exposure (ANOVA *p* < 0.1) although there was a sex^∗^treatment interaction (ANOVA interaction effect *p* = 0.024, Supplementary Figure 4A). A similar result was obtained for cytochrome oxidase (sex ^∗^ treatment = 0.079, Supplementary Figure 4B). The were similar trends in locomotor coordination and motor learning abilities of the animals. In the second trial, the time to perform the test was significantly decreased in controls when compared to the first trial, (*p* < 0.05), showing animals learned the task, that became a trend in the 22 ng/kg/day α-HBCDD-exposed animals (*p* = 0.09) and there was no difference at the highest dose (Supplementary Figure 4C), confirming that α-HBCDD exposure induced learning deficits, and the same pattern was observed for the number of missteps, confirming that motor deficits were induced.

## Discussion

Epigenetic modifications are known to occur in RNA, DNA and histones and, although they may change the accessibility of the DNA region and/or gene expression, the original DNA sequence remains unaltered (Robin, 1989). To date, 5mC was the ex libris of DNA methylation in eukaryotes. Only recently, 6mA, originally described in prokaryotes (Dunn and Smith, 1955, 1958) has been found in eukaryotes and proved to have functional roles (Wu et al., 2016; Yao et al., 2017, 2018; Xie et al., 2018). In this study, we demonstrate not only that in our laboratory 6mA is a genuine eukaryotic DNA modification, but also that it is widely distributed throughout rodents’ tissues, with major enrichment in the brain, as recently described (Yao et al., 2017). Furthermore, in a neurodevelopmental toxicity model, the trend in 6mA levels was influenced by perinatal exposure to the pollutant α-HBCDD and associated with the behavioral anomalies seen upto 8 months after exposure.

There is currently some uncertainty in the literature as to whether 6mA is a genuine eukaryotic DNA modification. Concerns have surrounded potential bacterial contamination, RNA presence in DNA readouts and antibody cross-reactivity (Ratel et al., 2006; O’Brown et al., 2019; Douvlataniotis et al., 2020). Initially, we confirmed that the methylation machinery is common to many eukaryotic species and that the available 3rd generation sequencing datasets provide direct-read evidence for the modification being found in regions of identifiable eukaryotic DNA. Furthermore, online available data from GTEX allowed us to explore the body expression levels of the different methylases and methyltransferases involved in the 6mA machinery. Recently, N6AMT1 was identified as a methyltransferase for 6mA and ALKBH1, a demethylase already shown to be present in mice (Wu et al., 2016), were expressed in humans (Xiao et al., 2018). In the same study, Xiao et al. (2018) also showed that manipulation of these enzymes directly affected the expression of 6mA, particularly in cancer cell lines. Knowledge of tissue-specific expression of these enzymes, as well as the methyltransferase METLL4, leads us to hypothesize that, in normal individuals, levels of 6mA are homeostatically maintained. Expression of these enzymes throughout the human body appear to be similar as we can see in Figure 2 and in the correlation graph from the Supplementary Material. Differences in 6-methyl adenine levels after stressors might be justified by an imbalance in the expression of such enzymes and their manipulation might help attenuate the outcome.

As potential sources of contamination have previously been reported (O’Brown et al., 2019), we took extra care when handling samples and extracting DNA: we removed the zebrafish chorion prior to DNA extraction, reducing the contamination from bacterial DNA, and as such, the 6mA levels represents that in the uncontaminated zebrafish gDNA. Similarly, mouse embryos were extracted from the sterile *in utero* environment and the DNA treated with RNAse, reducing the risk of bacterial and RNA contamination. We also extracted the genomic locations of 6mA from the MethSMRT database. This is an extraction of 6mA calls from direct long reads from PacBio Sequencing, with base-calling that identifies 6mA from the original read. This excludes the hypothesis that 6mA calls are a contaminant as the surrounding eukaryotic sequence is read, confirming the species of origin of 6mA. Furthermore, we demonstrated the concordance of the results of 6mA measurement by two methods (dot blot and LC-MS/MS), which leads us to believe that these two independent techniques are robust enough to allow the detection and quantification of 6mA. Although dot blots might present some limitations in regards to full quantification of this modification, LC-MS/MS completely erases any doubts that may exist. Has previously reported (Liu et al., 2016; Wu et al., 2016; Xiao et al., 2018; O’Brown et al., 2019), levels of 6mA are accurately detected and show no contamination of others adenine modifications (Xiao et al., 2018). Furthermore, levels of 6mA detected with LC-MS/MS, dot blot and SMRT-sequencing were shown to be similar in other reports (Wu et al., 2016; Xiao et al., 2018) and in our own data. Positive and negative controls for this modification are yet to be described and for that reason, we have decided to develop two of our own. Both in lab methylated yeast and oligonucleotide provided good quality positive controls as we can clearly see an increase in signal intensity after enzymatic treatment. However, these controls fail to give an exact measure of the numbers of adenines present in the sequence vs. number of adenine methylated.

Moreover, we were able to show, for the first time, that like 5mC, 6mA is present in tissues from all the major eukaryotic organ systems in mice, specifically in the digestive, cardiovascular and immune system. We also provide the first evidence of the presence of 6mA in twenty-eight different human brain regions. Interestingly, the brain region where we measured the higher levels of methylated adenine was the white matter, shown and described to take a great part on the development of the brain tumors (Louis et al., 2007; Esmaeili et al., 2018). These results, together with the RNA sequencing results illustrating the lower levels of the demethylases expression in the human brain, are in line with the recently described high levels of 6mA in Glioblastoma cell lines (Xie et al., 2018) and may help to elucidate the mechanisms behind such events. Furthermore, the role of the conserved methyltransferase and demethylase in the folate cycle and DNA methylation warrants further biochemical investigation. Increasing evidence highlights that chronical stress, particularly in early life, could interfere and regulate the levels of DNA methylation, which, in turn, acts on the expression of certain genes (Murgatroyd et al., 2009; Uchida et al., 2011; Vidrascu et al., 2019). Our results from animals exposed to α-HBCDD during early life are consistent with alterations in DNA methylation, with decreased levels of 6mA in the cerebellum, revealing the importance of this modification in the processes initiated by stress.

Earlier studies detected the accumulation of 6mA during embryogenesis, in genomic DNA from sperm, oocytes, and several embryonic stages of pig and zebrafish (Liu et al., 2016). Considering our previous results and the knowledge from embryogenesis, we decided to investigate the behavior of 6mA during the whole gestation period of zebrafish and mice. Our data from zebrafish embryos confirms the accumulation of methylated adenines during this period and, contrarily to what was previously published by Liu et al. (2016) we demonstrate that this modification steadily increases until birth. Recent data confirms this increase during development (Li et al., 2020). The levels of 6mA in the hippocampus of mice embryos provided similar results to the zebrafish, encouraging the hypothesis that 6mA has a role in neurogenesis. Opposite of what happens with 5mC that changes dynamically throughout embryogenesis (Greenberg and Bourc’his, 2019), 6-methyladenine appears to significantly change its levels from early embryonic days up to the very end of gestation. DNA methylation, namely 5mC or 5hmC, has already been proven to play an important role in neurodevelopment (Ficz et al., 2011; Szulwach et al., 2011; Wang et al., 2016) and deviations to a normal pregnancy, in various forms, led to neurodevelopmental disorders (Chen et al., 2013; Kundakovic and Jaric, 2017; Palma-Gudiel et al., 2019). The physiological role of 6mA appears to be in the silencing of both genes and transposons such as at long interspersed element 1 (LINE-1) (Li et al., 2020). Although LINE-1 retrotransposons make up around 17% of the human genome, they are highly mobile, and cause somatic mosaicism that has been linked to both neurodevelopmental disorders and psychiatric disorders such as Schizophrenia (Bundo et al., 2014; Doyle et al., 2017).

Finally, changes in 6mA level in cerebellum were measured at PND270 in a rodent model that was shown to concurrently induce neurobehavioral deficiencies (Maurice et al., 2015). Indeed, the neurodevelopmental toxicity of α-HBCDD was recently studied by evaluating neurobehavioral impairments induced by α-HBCDD exposure via the food during the gestation and lactation in dam rats at concentration levels which were representative of human exposure (22 and 66 ng/kg/day) (Sengupta, 2013). During the first 3 weeks of life, impairments in motor maturation of pups were observed in a dose-dependent manner depending on the test, whereas no significant differences were reported between male and female pups. At PND26, the anxiety levels of female rats exposed to the lowest dose of α-HBCDD (22 ng/kg/day) was significantly reduced whereas it remained unchanged in males. No significant *v*ariations were measured in rats exposed to the higher level of the pollutant (66 ng/kg/day). These observations are in line with the observed changes in DNA methylation (slight decrease in 6mA levels in the cerebellum) and CO activity (slight decrease measured in the interpositus nucleus). However, alterations in locomotor coordination and learning capabilities observed in the same animals later in life, at PND270, appear to be inversely proportional to 6mA levels, suggesting a potential role of 6mA epigenetic change in the brain toxicity initiated by α-HBCDD exposure. Further investigations need to be carried out to confirm if early life exposure to α-HBCDD may induce changes in 6mA epigenetic hallmarks in the developing brain, which in turn may affect behavior of the offspring at adulthood.

To conclude, our observation that 6mA levels continuously increase throughout the whole prenatal development suggests that this modification is introduced as the fetus differentiates and fully differentiated somatic cells accumulate. As such, the role of 6mA in the developmental origins of health and disease is obvious. Given the developmental role that is becoming apparent in the literature, we have expanded this. We report that 6mA steadily increases during embryogenesis in both zebrafish and mice and its global levels show subtle changes in the cerebellum of female rats upon exposure to brominated flame retardant during early life. Nevertheless, further studies are required in order to better understand the role and plasticity of 6mA in development and pathophysiological processes. Our findings suggest that the link between 6-methyladenine, neurogenesis, and changes in behavior as well as inflammation in the adult brain is worth exploring in more detail in the future.

## Data Availability Statement

The raw data supporting the conclusions of this article will be made available by the authors, without undue reservation.

## Ethics Statement

The animal study was reviewed and approved by the Institutional Ethics Committee, University of Lorraine, LIH Institutional Animal Welfare structure requirements as well as the European Union Directive 2010/63/EU as implemented in National Legislation.

## Author Contributions

SF and JT: conceptualization and literature review. SF, SR, NG, SM, RD, LG, and HS: data collection. AL, IE, and YD: provide samples. SF, SR, NG, HS, and JT: data analysis. SF, NG, and JT: manuscript writing and editing. All authors read and approved the final version of the manuscript.

## Conflict of Interest

The authors declare that the research was conducted in the absence of any commercial or financial relationships that could be construed as a potential conflict of interest.

## Supplementary Material

Supplementary Figure 1 Representation of the linearity of expression levels for methylase and demethylase enzymes. **(A)** ALKBH1 vs. METLL4; **(B)** ALKBH1 vs. N6AMT1. Data from the GTEX portal on August 19th, (dbGaP Accession phs000424.v8.p2, 19/08/2020) (linear relationship was evaluated by Pearson analysis, R2 given in each panel, *p* < 0.01 in both cases).

Supplementary Figure 2 **(A)** Genotype-Tissue Expression data for human N6AMT1. Data were downloaded from the GTEX portal on August 19th, (dbGaP Accession phs000424.v8.p2, 19/08/2020). **(B)** Hierarchical clustering of sequence alignment and percentage sequence similarity for the methyl transferase N6AMT1.

Supplementary Figure 3 **(A)** Representative membranes for dot-blot quantification of the artificially methylated DNA control to demonstrate the lack of binding in synthetic DNA without 6mA and the strong signal from the artificially modified DNA. **(B)** Spike-in oligo sequence and sanger sequence confirmation.

## Abbreviations

6mA: 6-methyladenine
CO: cytochrome c oxidase
HPF: hours post fertilization
HBCDD: HexaBromoCycloDoDecane
LC-MS/MS: liquid chromatography tandem mass spectroscopy
PND: post-natal day.

## References

[B1] Alvarado-Cruz I. Alegria-Torres J. A. Montes-Castro N. Jimenez-Garza O. Quintanilla-Vega B. (2018). Environmental epigenetic changes, as risk factors for the development of diseases in children: a systematic review. *Ann. Glob. Health* 84 212–224. 10.29024/aogh.909 30873799 PMC6748183

[B2] Bonsch D. Wunschel M. Lenz B. Janssen G. Weisbrod M. Sauer H. (2012). Methylation matters? Decreased methylation status of genomic DNA in the blood of schizophrenic twins. *Psychiatry Res.* 198 533–537. 10.1016/j.psychres.2011.09.004 23102571

[B3] Bundo M. Toyoshima M. Okada Y. Akamatsu W. Ueda J. Nemoto-Miyauchi T. (2014). Increased l1 retrotransposition in the neuronal genome in schizophrenia. *Neuron* 81 306–313. 10.1016/j.neuron.2013.10.053 24389010

[B4] Cao-Lei L. Suwansirikul S. Jutavijittum P. Meriaux S. B. Turner J. D. Muller C. P. (2013). Glucocorticoid receptor gene expression and promoter CpG modifications throughout the human brain. *J. Psychiatr. Res.* 47 1597–1607. 10.1016/j.jpsychires.2013.07.022 23948638

[B5] Cardenas A. Rifas-Shiman S. L. Godderis L. Duca R. C. Navas-Acien A. Litonjua A. A. (2017). Prenatal exposure to mercury: associations with global DNA methylation and hydroxymethylation in cord blood and in childhood. *Environ. Health Perspect.* 125:087022. 10.1289/EHP1467 28934725 PMC5783674

[B6] Chen Y. Ozturk N. C. Zhou F. C. (2013). DNA methylation program in developing hippocampus and its alteration by alcohol. *PLoS One* 8:e60503. 10.1371/journal.pone.0060503 23544149 PMC3609790

[B7] Crepeaux G. Grova N. Bouillaud-Kremarik P. Sikhayeva N. Salquebre G. Rychen G. (2014). Short-term effects of a perinatal exposure to a 16 polycyclic aromatic hydrocarbon mixture in rats: assessment of early motor and sensorial development and cerebral cytochrome oxidase activity in pups. *Neurotoxicology* 43 90–101. 10.1016/j.neuro.2014.03.012 24709092

[B8] De Nys S. Duca R. C. Nawrot T. Hoet P. Van Meerbeek B. Van Landuyt K. L. (2018). Temporal variability of global DNA methylation and hydroxymethylation in buccal cells of healthy adults: association with air pollution. *Environ. Int.* 111 301–308. 10.1016/j.envint.2017.11.002 29217223

[B9] Douvlataniotis K. Bensberg M. Lentini A. Gylemo B. Nestor C. E. (2020). No evidence for DNA N (6)-methyladenine in mammals. *Sci. Adv.* 6:eaay3335. 10.1126/sciadv.aay3335 32206710 PMC7080441

[B10] Doyle G. A. Crist R. C. Karatas E. T. Hammond M. J. Ewing A. D. Ferraro T. N. (2017). Analysis of LINE-1 elements in DNA from postmortem brains of individuals with schizophrenia. *Neuropsychopharmacology* 42 2602–2611. 10.1038/npp.2017.115 28585566 PMC5686486

[B11] Duca R. C. Grova N. Ghosh M. Do J. M. Hoet P. H. M. Vanoirbeek J. A. J. (2018). Exposure to polycyclic aromatic hydrocarbons leads to non-monotonic modulation of DNA and RNA (hydroxy)methylation in a rat model. *Sci. Rep.* 8:10577. 30002487 10.1038/s41598-018-28911-y PMC6043565

[B12] Dunn D. B. Smith J. D. (1955). Occurrence of a new base in the deoxyribonucleic acid of a strain of bacterium coli. *Nature* 175 336–337. 10.1038/175336a0 13235889

[B13] Dunn D. B. Smith J. D. (1958). The occurrence of 6-methylaminopurine in deoxyribonucleic acids. *Biochem. J.* 68 627–636. 10.1042/bj0680627 13522672 PMC1200409

[B14] Elwenspoek M. M. C. Hengesch X. Leenen F. A. D. Sias K. Fernandes S. B. Schaan V. K. (2019). Glucocorticoid receptor signaling in leukocytes after early life adversity. *Dev. Psychopathol.* 32 853–863. 10.1017/s0954579419001147 31407649

[B15] Ernens I. Lumley A. I. Zhang L. Devaux Y. Wagner D. R. (2017). Hypoxia inhibits lymphatic thoracic duct formation in zebrafish. *Biochem. Biophys. Res. Commun.* 482 1129–1134. 10.1016/j.bbrc.2016.11.169 27916465

[B16] Esmaeili M. Stensjøen A. L. Berntsen E. M. Solheim O. Reinertsen I. (2018). The direction of tumour growth in glioblastoma patients. *Sci. Rep.* 8:1199. 29352231 10.1038/s41598-018-19420-z PMC5775193

[B17] Ficz G. Branco M. R. Seisenberger S. Santos F. Krueger F. Hore T. A. (2011). Dynamic regulation of 5-hydroxymethylcytosine in mouse ES cells and during differentiation. *Nature* 473 398–402. 10.1038/nature10008 21460836

[B18] Godderis L. Schouteden C. Tabish A. Poels K. Hoet P. Baccarelli A. A. (2015). Global methylation and hydroxymethylation in DNA from blood and saliva in healthy volunteers. *Biomed. Res. Int.* 2015:845041. 10.1155/2015/845041 26090450 PMC4450276

[B19] Greenberg M. V. C. Bourc’his D. (2019). The diverse roles of DNA methylation in mammalian development and disease. *Nat. Rev. Mol. Cell Biol.* 20 590–607. 10.1038/s41580-019-0159-6 31399642

[B20] Grova N. Schroeder H. Olivier J. L. Turner J. D. (2019). Epigenetic and neurological impairments associated with early life exposure to persistent organic pollutants. *Int. J. Genomics* 2019:2085496. 30733955 10.1155/2019/2085496 PMC6348822

[B21] Grova N. Wang X. Hardy E. M. Palazzi P. Chata C. Appenzeller B. M. R. (2020). Ultra performance liquid chromatography - tandem mass spectrometer method applied to the analysis of both thyroid and steroid hormones in human hair. *J. Chromatogr. A* 1612:460648. 10.1016/j.chroma.2019.460648 31679711

[B22] Ibhazehiebo K. Iwasaki T. Xu M. Shimokawa N. Koibuchi N. (2011). Brain-derived neurotrophic factor (BDNF) ameliorates the suppression of thyroid hormone-induced granule cell neurite extension by hexabromocyclododecane (HBCD). *Neurosci. Lett.* 493 1–7. 10.1016/j.neulet.2011.01.062 21281695

[B23] Kigar S. L. Chang L. Guerrero C. R. Sehring J. R. Cuarenta A. Parker L. L. (2017). N6-methyladenine is an epigenetic marker of mammalian early life stress. *Sci. Rep.* 7:18078. 29273787 10.1038/s41598-017-18414-7 PMC5741724

[B24] Koch C. Schmidt-Kotters T. Rupp R. Sures B. (2015). Review of hexabromocyclododecane (HBCD) with a focus on legislation and recent publications concerning toxicokinetics and -dynamics. *Environ. Pollut.* 199 26–34. 10.1016/j.envpol.2015.01.011 25618363

[B25] Koziol M. J. Bradshaw C. R. Allen G. E. Costa A. S. H. Frezza C. Gurdon J. B. (2016). Identification of methylated deoxyadenosines in vertebrates reveals diversity in DNA modifications. *Nat. Struct. Mol. Biol.* 23 24–30. 10.1038/nsmb.3145 26689968 PMC4941928

[B26] Kundakovic M. Jaric I. (2017). The Epigenetic Link between Prenatal Adverse Environments and Neurodevelopmental Disorders. *Genes* 8:104. 10.3390/genes8030104 28335457 PMC5368708

[B27] Kweon S. M. Chen Y. Moon E. Kvederaviciute K. Klimasauskas S. Feldman D. E. (2019). An adversarial DNA N(6)-methyladenine-sensor network preserves polycomb silencing. *Mol. Cell.* 74 1138.e1136–1147.e1136. 30982744 10.1016/j.molcel.2019.03.018 PMC6591016

[B28] LaSalle J. M. (2011). A genomic point-of-view on environmental factors influencing the human brain methylome. *Epigenetics* 6 862–869. 10.4161/epi.6.7.16353 21617367 PMC3154427

[B29] Li X. Zhao Q. Wei W. Lin Q. Magnan C. Emami M. R. (2019). The DNA modification N6-methyl-2’-deoxyadenosine (m6dA) drives activity-induced gene expression and is required for fear extinction. *Nat. Neurosci.* 22 534–544. 10.1038/s41593-019-0339-x 30778148 PMC6462436

[B30] Li Z. Zhao S. Nelakanti R. V. Lin K. Wu T. P. Alderman M. H. (2020). N(6)-methyladenine in DNA antagonizes SATB1 in early development. *Nature* 583 625–630. 10.1038/s41586-020-2500-9 32669713 PMC8596487

[B31] Liu J. Zhu Y. Luo G.-Z. Wang X. Yue Y. Wang X. (2016). Abundant DNA 6mA methylation during early embryogenesis of zebrafish and pig. *Nat. Commun.* 7:13052. 27713410 10.1038/ncomms13052 PMC5059759

[B32] Louis D. N. Ohgaki H. Wiestler O. D. Cavenee W. K. Burger P. C. Jouvet A. (2007). The 2007 WHO classification of tumours of the central nervous system. *Acta Neuropathol.* 114 97–109. 10.1007/978-94-007-1399-4_10 17618441 PMC1929165

[B33] Low D. A. Casadesus J. (2008). Clocks and switches: bacterial gene regulation by DNA adenine methylation. *Curr. Opin. Microbiol.* 11 106–112. 10.1016/j.mib.2008.02.012 18396448

[B34] Madeira F. Park Y. M. Lee J. Buso N. Gur T. Madhusoodanan N. (2019). The EMBL-EBI search and sequence analysis tools APIs in 2019. *Nucleic Acids Res.* 47 W636–W641. 30976793 10.1093/nar/gkz268 PMC6602479

[B35] Marinus M. G. Casadesus J. (2009). Roles of DNA adenine methylation in host-pathogen interactions: mismatch repair, transcriptional regulation, and more. *FEMS Microbiol. Rev.* 33 488–503. 10.1111/j.1574-6976.2008.00159.x 19175412 PMC2941194

[B36] Maurice N. Olry J. C. Cariou R. Dervilly-Pinel G. Le Bizec B. Travel A. (2015). Short-term effects of a perinatal exposure to the HBCDD alpha-isomer in rats: assessment of early motor and sensory development, spontaneous locomotor activity and anxiety in pups. *Neurotoxicol. Teratol.* 52 170–180. 10.1016/j.ntt.2015.08.005 26348671

[B37] Mitchell C. Schneper L. M. Notterman D. A. (2016). DNA methylation, early life environment, and health outcomes. *Pediatr. Res.* 79 212–219. 10.1038/pr.2015.193 26466079 PMC4798238

[B38] Murgatroyd C. Patchev A. V. Wu Y. Micale V. Bockmuhl Y. Fischer D. (2009). Dynamic DNA methylation programs persistent adverse effects of early-life stress. *Nat. Neurosci.* 12 1559–1566. 10.1038/nn.2436 19898468

[B39] Murphy T. M. O’donovan A. Mullins N. O’farrelly C. Mccann A. Malone K. (2015). Anxiety is associated with higher levels of global DNA methylation and altered expression of epigenetic and interleukin-6 genes. *Psychiatr. Genet.* 25 71–78. 10.1097/ypg.0000000000000055 25350786

[B40] O’Brown Z. K. Boulias K. Wang J. Wang S. Y. O’brown N. M. Hao Z. (2019). Sources of artifact in measurements of 6mA and 4mC abundance in eukaryotic genomic DNA. *BMC Genomics* 20:445. 10.1186/s12864-019-5754-6 31159718 PMC6547475

[B41] Palma-Gudiel H. Eixarch E. Crispi F. Moran S. Zannas A. S. Fananas L. (2019). Prenatal adverse environment is associated with epigenetic age deceleration at birth and hypomethylation at the hypoxia-responsive EP300 gene. *Clin. Epigenetics* 11 73. 31072398 10.1186/s13148-019-0674-5 PMC6507133

[B42] Papadopoulos J. S. Agarwala R. (2007). COBALT: constraint-based alignment tool for multiple protein sequences. *Bioinformatics* 23 1073–1079. 10.1093/bioinformatics/btm076 17332019

[B43] Ratel D. Ravanat J. L. Charles M. P. Platet N. Breuillaud L. Lunardi J. (2006). Undetectable levels of N6-methyl adenine in mouse DNA: cloning and analysis of PRED28, a gene coding for a putative mammalian DNA adenine methyltransferase. *FEBS Lett.* 580 3179–3184. 10.1016/j.febslet.2006.04.074 16684535

[B44] Robin H. (1989). DNA methylation and epigenetic mechanisms. *Cell Biophys.* 15 15–20. 10.1007/bf02991575 2476223

[B45] Roseboom T. J. Painter R. C. Van Abeelen A. F. Veenendaal M. V. De Rooij S. R. (2011). Hungry in the womb: what are the consequences? Lessons from the Dutch famine. *Maturitas* 70 141–145. 10.1016/j.maturitas.2011.06.017 21802226

[B46] Sanchez-Romero M. A. Cota I. Casadesus J. (2015). DNA methylation in bacteria: from the methyl group to the methylome. *Curr. Opin. Microbiol.* 25 9–16. 10.1016/j.mib.2015.03.004 25818841

[B47] Schiffers S. Ebert C. Rahimoff R. Kosmatchev O. Steinbacher J. Bohne A. V. (2017). Quantitative LC-MS provides no evidence for m(6) dA or m(4) dC in the genome of mouse embryonic stem cells and tissues. *Angew. Chem. Int. Ed. Engl.* 56 11268–11271. 10.1002/anie.201700424 28371147

[B48] Schneider C. A. Rasband W. S. Eliceiri K. W. (2012). NIH Image to ImageJ: 25 years of image analysis. *Nat. Methods* 9 671–675. 10.1038/nmeth.2089 22930834 PMC5554542

[B49] Sengupta P. (2013). The laboratory rat: relating its age with human’s. *Int. J. Prev. Med.* 4 624–630. 23930179 PMC3733029

[B50] Szulwach K. E. Li X. Li Y. Song C. X. Wu H. Dai Q. (2011). 5-hmC-mediated epigenetic dynamics during postnatal neurodevelopment and aging. *Nat. Neurosci.* 14 1607–1616. 10.1038/nn.2959 22037496 PMC3292193

[B51] Tobi E. W. Slieker R. C. Luijk R. Dekkers K. F. Stein A. D. Xu K. M. (2018). DNA methylation as a mediator of the association between prenatal adversity and risk factors for metabolic disease in adulthood. *Sci. Adv.* 4:eaao4364. 10.1126/sciadv.aao4364 29399631 PMC5792223

[B52] Trivedi M. S. Abreu M. M. Sarria L. Rose N. Ahmed N. Beljanski V. (2019). Alterations in DNA methylation status associated with gulf war illness. *DNA Cell Biol.* 38 561–571. 10.1089/dna.2018.4469 30920300

[B53] Uchida S. Hara K. Kobayashi A. Otsuki K. Yamagata H. Hobara T. (2011). Epigenetic status of Gdnf in the ventral striatum determines susceptibility and adaptation to daily stressful events. *Neuron* 69 359–372. 10.1016/j.neuron.2010.12.023 21262472

[B54] Vidrascu E. M. Bashore A. C. Howard T. D. Moore J. B. (2019). Effects of early- and mid-life stress on DNA methylation of genes associated with subclinical cardiovascular disease and cognitive impairment: a systematic review. *BMC Med. Genet.* 20:39. 10.1186/s12881-019-0764-4 30866842 PMC6417232

[B55] Vonderwalde I. (2019). DNA methylation within the amygdala early in life increases susceptibility for depression and anxiety disorders. *J. Neurosci.* 39:8828. 10.1523/jneurosci.0845-19.2019 31694977 PMC6832684

[B56] Wang Z. Tang B. He Y. Jin P. (2016). DNA methylation dynamics in neurogenesis. *Epigenomics* 8 401–414. 10.2217/epi.15.119 26950681 PMC4864063

[B57] Wion D. Casadesús J. (2006). N6-methyl-adenine: an epigenetic signal for DNA-protein interactions. *Nat. Rev. Microbiol.* 4 183–192. 10.1038/nrmicro1350 16489347 PMC2755769

[B58] Wu T. P. Wang T. Seetin M. G. Lai Y. Zhu S. Lin K. (2016). DNA methylation on N(6)-adenine in mammalian embryonic stem cells. *Nature* 532 329–333. 10.1038/nature17640 27027282 PMC4977844

[B59] Xiao C. L. Zhu S. He M. Chen D. Zhang Q. Chen Y. (2018). N(6)-methyladenine DNA modification in the human genome. *Mol. Cell.* 71 306.e307–318.e307. 30017583 10.1016/j.molcel.2018.06.015

[B60] Xie Q. Wu T. P. Gimple R. C. Li Z. Prager B. C. Wu Q. (2018). N(6)-methyladenine DNA modification in Glioblastoma. *Cell* 175 1228.e1120–1243.e1120. 30392959 10.1016/j.cell.2018.10.006 PMC6433469

[B61] Yao B. Cheng Y. Wang Z. Li Y. Chen L. Huang L. (2017). DNA N6-methyladenine is dynamically regulated in the mouse brain following environmental stress. *Nat. Commun.* 8:1122. 29066820 10.1038/s41467-017-01195-y PMC5654764

[B62] Yao B. Li Y. Wang Z. Chen L. Poidevin M. Zhang C. (2018). Active N(6)-methyladenine demethylation by DMAD regulates gene expression by coordinating with polycomb protein in neurons. *Mol. Cell.* 71 848.e846–857.e846. 30078725 10.1016/j.molcel.2018.07.005 PMC6136845

[B63] Ye P. Luan Y. Chen K. Liu Y. Xiao C. Xie Z. (2017). MethSMRT: an integrative database for DNA N6-methyladenine and N4-methylcytosine generated by single-molecular real-time sequencing. *Nucleic Acids Res.* 45 D85–D89. 27924023 10.1093/nar/gkw950 PMC5210644

[B64] Zhang G. Huang H. Liu D. Cheng Y. Liu X. Zhang W. (2015). N6-methyladenine DNA modification in *Drosophila*. *Cell* 161 893–906. 25936838 10.1016/j.cell.2015.04.018

[B65] Zhou C. Wang C. Liu H. Zhou Q. Liu Q. Guo Y. (2018). Identification and analysis of adenine N(6)-methylation sites in the rice genome. *Nat. Plants* 4 554–563. 10.1038/s41477-018-0214-x 30061746

