# Supplementary material for: N6-Methyladenine in Eukaryotic DNA: Tissue Distribution, Early Embryo Development, and Neuronal Toxicity
Source: Front Genet. 2021 May 24;12:657171. doi: 10.3389/fgene.2021.657171 (PMC8181416; doi:10.3389/fgene.2021.657171)

N6-methyladenine in eukaryotic DNA: tissue distribution, early embryo development and neuronal toxicity

Sara B. Fernandes [1,2], Nathalie Grova [1,3], Sarah Roth [1], Radu-Corneliu Duca [4,5], Lode Godderis [5,6], Pauline Guebels [1], Sophie B. Mériaux [1], Andrew I. Lumley [7], P. Bouillaud [3], Isabelle Ernens [7], Yvan Devaux [7], Henri Schroeder [3] and Jonathan D. Turner [1,*]

1 Immune Endocrine Epigenetics Research Group, Department of Infection and Immunity, Luxembourg Institute of Health, Esch-sur-Alzette, Luxembourg; SaraBeatriz.Fernandes@lih.lu; Nathalie.Grova@lih.lu; Sarah.Roth@lih.lu; Sophie.Meriaux@lih.lu
2 Faculty of Science, University of Luxembourg, Belval, Luxembourg;
3 Calbinotox, EA7488, Faculty of Science and Technology, Lorraine University, Vandoeuvre-lès Nancy, France; Henri.Schroeder@univ-lorraine.fr
4 Unit Environmental Hygiene and Human Biological Monitoring, Department of Health Protection, National Health Laboratory (LNS), Dudelange, Luxembourg; Radu.DUCA@lns.etat.lu
5 Centre for Environment and Health, Department of Public Health and Primary care, KU Leuven, Leuven, Belgium
6 Idewe, External Service for Prevention and Protection at work, Heverlee, Belgium
7 Cardiovascular Research Unit, Department of Public Health, Luxembourg Institute of Health, Strassen, Luxembourg; Andrew.lumley@lih.lu; isabelle.ernens@lih.lu; Yvan.devaux@lih.lu

* Correspondence: jonathan.turner@lih.lu ; Tel.: +352 26 97 0629

SUP. FIG 1A and B: DOT BLOT AND LC-MS/MS - LINEARITY OF THE RESULTS BETWEEN ENZYMES

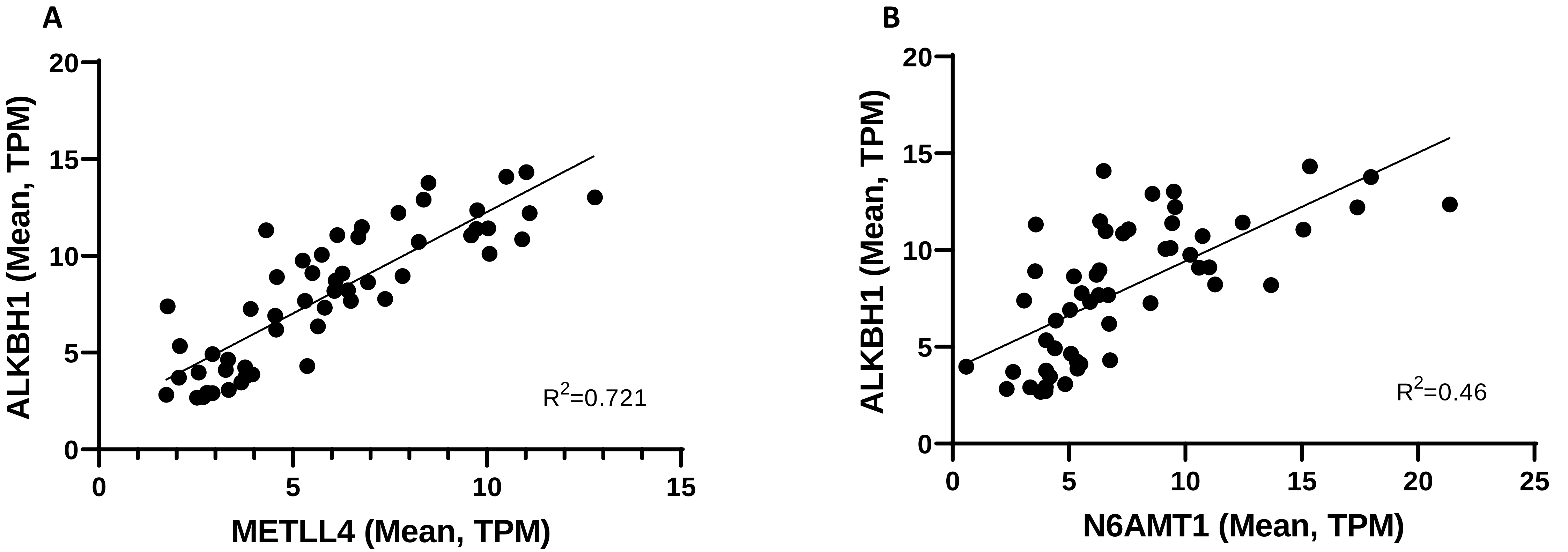

Representation of the linearity of expression levels for methylaseand demethylase enzymes. (A) ALKBH1 vs METLL4; (B) ALKBH1 vs N6AMT1. Data from the GTEX portal on August 19th, (dbGaPAccession phs000424.v8.p2, 19/08/2020) (linear relationship was evaluated by Pearson analysis, R2given in each panel, p< 0.01 in both cases).

SUP. FIG. 2A: TISSUE DISTRIBUTION OF N6AMT1

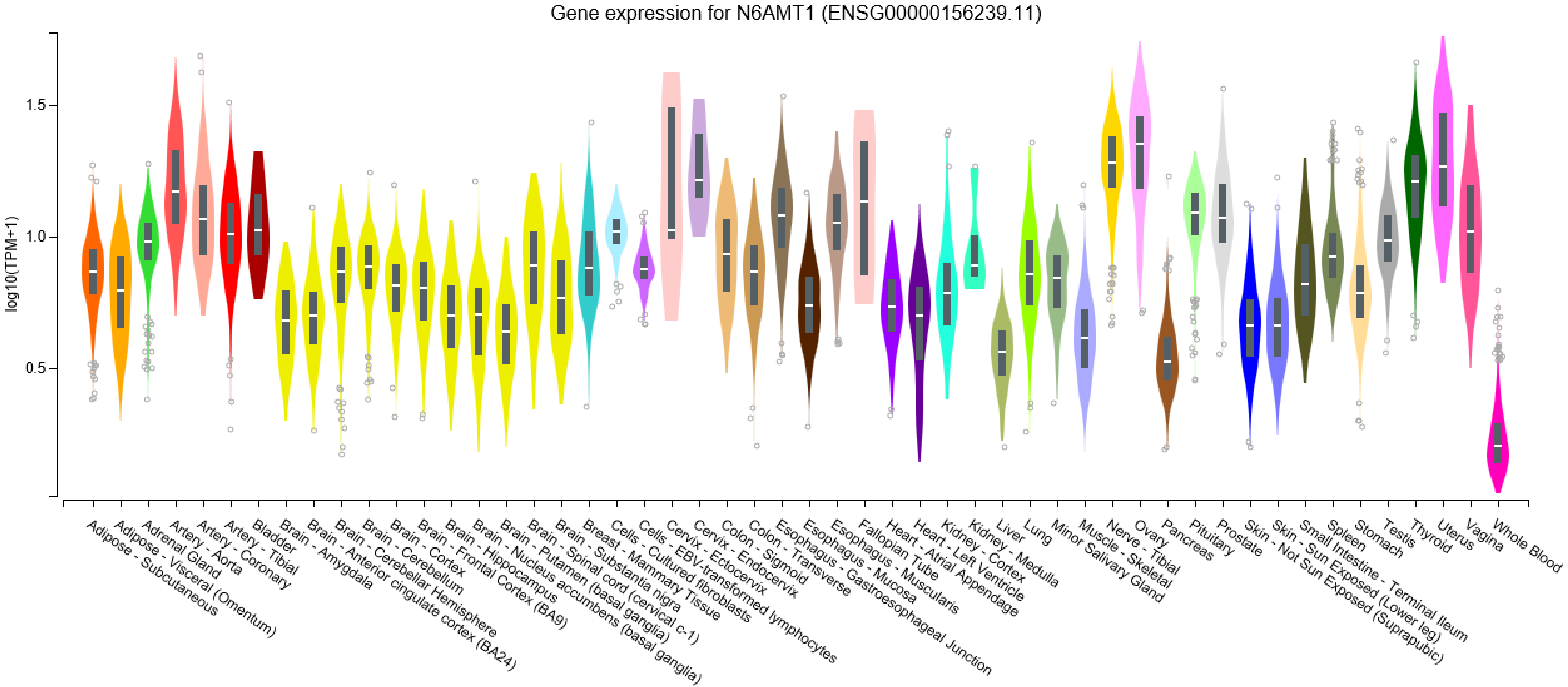

Genotype-Tissue Expression data for human N6AMT1. Data were downloaded from the GTEX portal on August 19th, (dbGaPAccession phs000424.v8.p2, 19/08/2020

SUP. FIG. 2B: Similarity Of N6AMT1

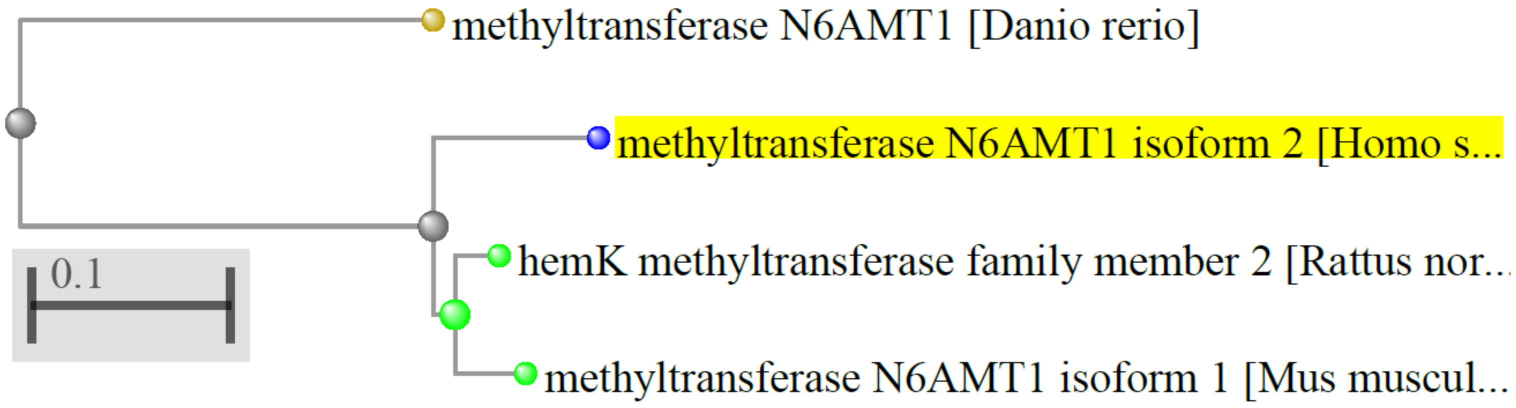

N6AMT1	% similarity
Homo Sapiens	100
Mus musculus	76.1
Rattus Norvegicus	77.5
Danio rerio	53

Hierarchical clustering of sequence alignment and percentage sequence similarity for the methyl transferase N6AMT1.

SUP. FIG. 3A: OLIGO POSITIVE CONTROL

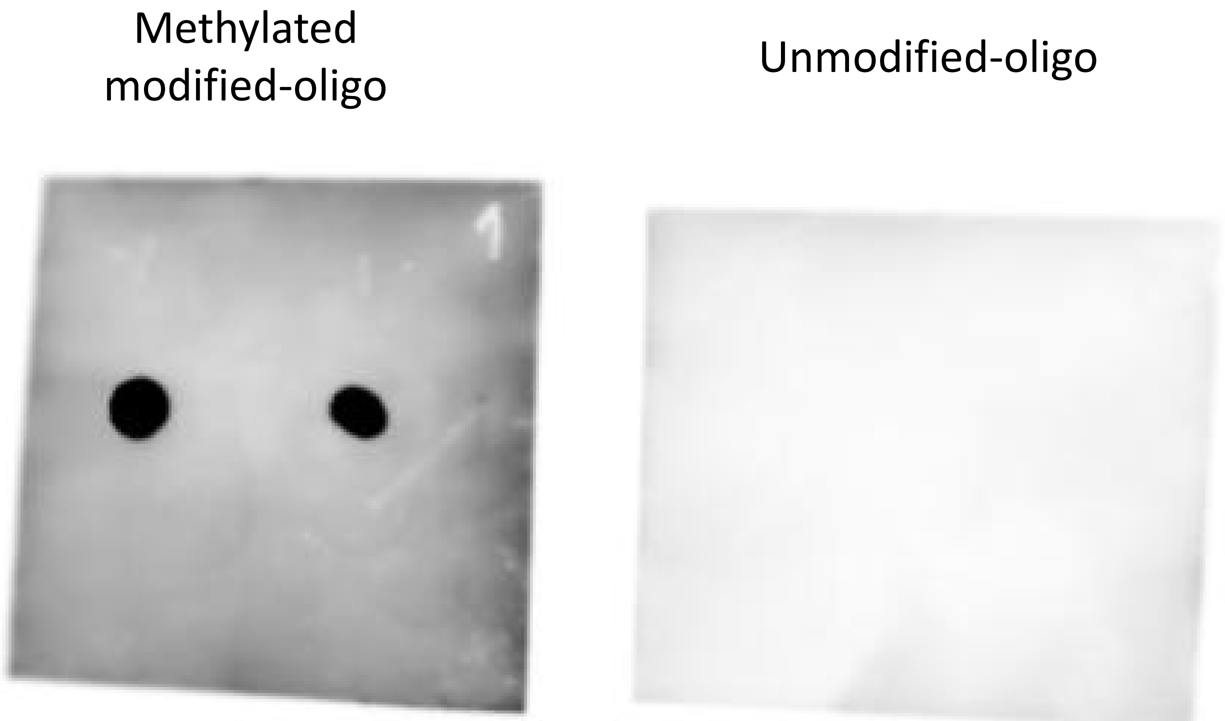

Representative membranes for dot-blot quantification of the artificially methylated DNA control to demonstrate the lack of binding in synthetic DNA without 6mA and the strong signal from the artificially modified DNA.

Sup.Fig.3B: Spike-in oligo sequence and sanger sequence confirmation

TCGCGCGTTTCGGTGATGACGGTGAAAACCTCTGACACATGCAGCTCCCGGAGACGGTCACAGCTTGTCTGTAAGCGGATGCCGGGAGCAGACAAGCCCGTCAGGGCGCGTCAGCGGGTGTTGG
CGGGTGTCGGGGCTGGCTTAACTATGCGGCATCAGAGCAGATTGTACTGAGAGTGCACCATATGCGGTGTGAAATACCGCACAGATGCGTAAGGAGAAAATACCGCATCAGGCGCCATTCGCCAT
TCAGGCTGCGCAACTGTTGGGAAGGGCGATCGGTGCGGGCCTCTTCGCTATTACGCCAGCTGGCGAAAGGGGGATGTGCTGCAAGGCGATTAAGTTGGGTAACGCCAGGGTTTTCCCAGTCACG
ACGTT*GTAAAACGACGGCCAGTGAATTCGAGCTCGGTACCTCGCGAATGCATCTAGAT*__*GCCTCGTGAAATCCCGTTAGTTCGTGATTCTAAAAGGATCGGTAAACCTGATCGTTGTACAAGATCTG*__
__*TTAGGAATTGATCAATGCTTATCTACGATCGCAAGTGATGATCTTACCAAAAGATCGTTTATTGAGATCGGACCCAGGATCTCTTTTCCAATGATCTATTGTTTCGTGATTCTGAAGGTGCCAAGA*__
__*AGTTTCC*__*ATCGGATCCCGGGCCCGTCGACTGCAGAGGCCTGCATGCAAGCTTGGCGTAATCATGGTCATAGCTGTTTCCTG*TGTGAAATTGTTATCCGCTCACAATTCCACACAACATACGAGCCGG
AAGCATAAAGTGTAAAGCCTGGGGTGCCTAATGAGTGAGCTAACTCACATTAATTGCGTTGCGCTCACTGCCCGCTTTCCAGTCGGGAAACCTGTCGTGCCAGCTGCATTAATGAATCGGCCAACG
CGCGGGGAGAGGCGGTTTGCGTATTGGGCGCTCTTCCGCTTCCTCGCTCACTGACTCGCTGCGCTCGGTCGTTCGGCTGCGGCGAGCGGTATCAGCTCACTCAAAGGCGGTAATACGGTTATCCAC
AGAATCAGGGGATAACGCAGGAAAGAACATGTGAGCAAAAGGCCAGCAAAAGGCCAGGAACCGTAAAAAGGCCGCGTTGCTGGCGTTTTTCCATAGGCTCCGCCCCCCTGACGAGCATCACAAA
AATCGACGCTCAAGTCAGAGGTGGCGAAACCCGACAGGACTATAAAGATACCAGGCGTTTCCCCCTGGAAGCTCCCTCGTGCGCTCTCCTGTTCCGACCCTGCCGCTTACCGGATACCTGTCCGCCT
TTCTCCCTTCGGGAAGCGTGGCGCTTTCTCATAGCTCACGCTGTAGGTATCTCAGTTCGGTGTAGGTCGTTCGCTCCAAGCTGGGCTGTGTGCACGAACCCCCCGTTCAGCCCGACCGCTGCGCCTT
ATCCGGTAACTATCGTCTTGAGTCCAACCCGGTAAGACACGACTTATCGCCACTGGCAGCAGCCACTGGTAACAGGATTAGCAGAGCGAGGTATGTAGGCGGTGCTACAGAGTTCTTGAAGTGGT
GGCCTAACTACGGCTACACTAGAAGAACAGTATTTGGTATCTGCGCTCTGCTGAAGCCAGTTACCTTCGGAAAAAGAGTTGGTAGCTCTTGATCCGGCAAACAAACCACCGCTGGTAGCGGTGGTT
TTTTTGTTTGCAAGCAGCAGATTACGCGCAGAAAAAAAGGATCTCAAGAAGATCCTTTGATCTTTTCTACGGGGTCTGACGCTCAGTGGAACGAAAACTCACGTTAAGGGATTTTGGTCATGAGATT
ATCAAAAAGGATCTTCACCTAGATCCTTTTAAATTAAAAATGAAGTTTTAAATCAATCTAAAGTATATATGAGTAAACTTGGTCTGACAGTTACCAATGCTTAATCAGTGAGGCACCTATCTCAGCGA
TCTGTCTATTTCGTTCATCCATAGTTGCCTGACTCCCCGTCGTGTAGATAACTACGATACGGGAGGGCTTACCATCTGGCCCCAGTGCTGCAATGATACCGCGAGACCCACGCTCACCGGCTCCAGAT
TTATCAGCAATAAACCAGCCAGCCGGAAGGGCCGAGCGCAGAAGTGGTCCTGCAACTTTATCCGCCTCCATCCAGTCTATTAATTGTTGCCGGGAAGCTAGAGTAAGTAGTTCGCCAGTTAATAGT
TTGCGCAACGTTGTTGCCATTGCTACAGGCATCGTGGTGTCACGCTCGTCGTTTGGTATGGCTTCATTCAGCTCCGGTTCCCAACGATCAAGGCGAGTTACATGATCCCCCATGTTGTGCAAAAAAG
CGGTTAGCTCCTTCGGTCCTCCGATCGTTGTCAGAAGTAAGTTGGCCGCAGTGTTATCACTCATGGTTATGGCAGCACTGCATAATTCTCTTACTGTCATGCCATCCGTAAGATGCTTTTCTGTGACTG
GTGAGTACTCAACCAAGTCATTCTGAGAATAGTGTATGCGGCGACCGAGTTGCTCTTGCCCGGCGTCAATACGGGATAATACCGCGCCACATAGCAGAACTTTAAAAGTGCTCATCATTGGAAAAC
GTTCTTCGGGGCGAAAACTCTCAAGGATCTTACCGCTGTTGAGATCCAGTTCGATGTAACCCACTCGTGCACCCAACTGATCTTCAGCATCTTTTACTTTCACCAGCGTTTCTGGGTGAGCAAAAACA
GGAAGGCAAAATGCCGCAAAAAAGGGAATAAGGGCGACACGGAAATGTTGAATACTCATACTCTTCCTTTTTCAATATTATTGAAGCATTTATCAGGGTTATTGTCTCATGAGCGGATACATATTTG
AATGTATTTAGAAAAATAAACAAATAGGGGTTCCGCGCACATTTCCCCGAAAAGTGCCACCTGACGTCTAAGAAACCATTATTATCATGACATTAACCTATAAAAATAGGCGTATCACGAGGCCCTT
TCGTC

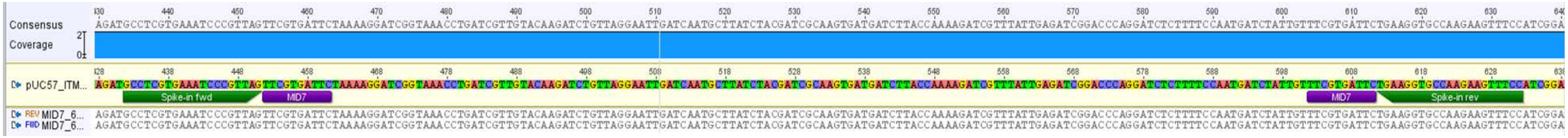

Spike-in oligo sequence and sanger sequence confirmation

Sup.Fig.4: Early life exposure to α-HBCDD induces change in 6mA levels in F1 generation at PND270

After demonstrating the steadily increase of 6mA throughout development, we decided to evaluate how values of 6mA change in the brain of rats followed by exposure to α-HBCDD through the dams in a known neurodevelopmental toxicity model. At PND270, the statistical analysis of 6mA levels in cerebellum showed an almost significant difference between the control and the animals treated with α-HBCDD, for both males and females (Supp. Fig 4A; ANOVA main effect p>0.05). Additionally, a significant interaction between sex and treatment (ANOVA interaction effect p=0.024) was observed, which can be explained by the decrease in 6mA observed at 22 ng/kg/day dose, only in females (Supp. Fig 4A). Upon measuring the cytochrome oxidase activity in the interpositus nucleus, the same interaction tendency was observed (sex * treatment = 0.079) (Supp. Fig 4B), which we also justify with the slight decrease at the lowest dose of α-HBCDD in females.
To confirm if the observed changes in cerebellar cells of female rats may result in behavioural impairments, we evaluated the locomotor coordination and motor learning abilities of the animals. The time spent in the apparatus and the number missteps were compared between the two trials. In the second trial, the time spent to perform the test was significantly decreased in controls when compared to the first one. The same tendency was observed between the two trials in the 22 ng/kg/day HBCDD-exposed animals (p=0.09) whereas no difference was observed at the highest dose (Supp. Fig 4C). Similarly, a slight decrease in the number of missteps between the two trials was also observed both in the controls (p=0.1) and the 22 ng/kg/day α-HBCDD group (p=0.06) but not at the highest dose (Supp. Fig 4D). These results suggest that perinatal exposure to α-HBCDD at 66 ng/kg/day may induce a deficit of coordination and motor learning skills in adult female rats, but 22ng/kg/day is not enough to induce such behavioral changes.

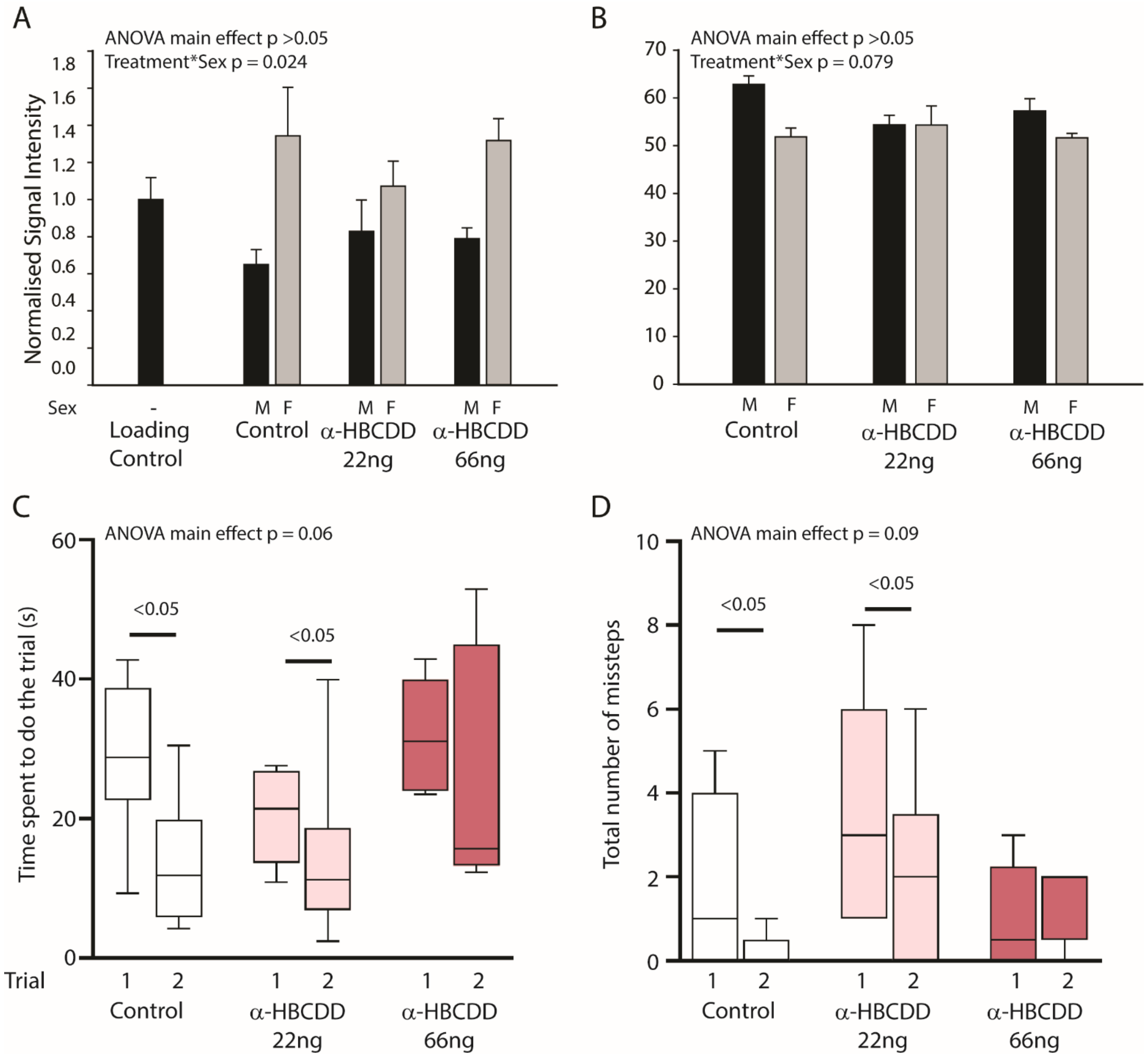

Supplement: Supplementary Figure 1 — Representation of the linearity of expression levels for methylase and demethylase enzymes. (A) ALKBH1 vs. METLL4; (B) ALKBH1 vs. N6AMT1. Data from the GTEX portal on August 19th, (dbGaP Accession phs000424.v8.p2, 19/08/2020) (linear relationship was evaluated by Pearson analysis, R2 given in each panel, p < 0.01 in both cases). [file Data_Sheet_1.PDF]
